# Spatial analysis and mapping of malaria risk in Malawi using point-referenced prevalence of infection data

**DOI:** 10.1186/1476-072X-5-41

**Published:** 2006-09-20

**Authors:** Lawrence N Kazembe, Immo Kleinschmidt, Timothy H Holtz, Brian L Sharp

**Affiliations:** 1 Mathematical Sciences Department, Chancellor College, University of Malawi, Zomba, Malawi; 2 Malaria Research Programme, Medical Research Council, Durban, South Africa; 3 International Research and Programs Branch, Division of Tuberculosis Elimination, Centers for Disease Control and Prevention, Altlanta, GA, USA

## Abstract

**Background:**

Current malaria control initiatives aim at reducing malaria burden by half by the year 2010. Effective control requires evidence-based utilisation of resources. Characterizing spatial patterns of risk, through maps, is an important tool to guide control programmes. To this end an analysis was carried out to predict and map malaria risk in Malawi using empirical data with the aim of identifying areas where greatest effort should be focussed.

**Methods:**

Point-referenced prevalence of infection data for children aged 1–10 years were collected from published and grey literature and geo-referenced. The model-based geostatistical methods were applied to analyze and predict malaria risk in areas where data were not observed. Topographical and climatic covariates were added in the model for risk assessment and improved prediction. A Bayesian approach was used for model fitting and prediction.

**Results:**

Bivariate models showed a significant association of malaria risk with elevation, annual maximum temperature, rainfall and potential evapotranspiration (PET). However in the prediction model, the spatial distribution of malaria risk was associated with elevation, and marginally with maximum temperature and PET. The resulting map broadly agreed with expert opinion about the variation of risk in the country, and further showed marked variation even at local level. High risk areas were in the low-lying lake shore regions, while low risk was along the highlands in the country.

**Conclusion:**

The map provided an initial description of the geographic variation of malaria risk in Malawi, and might help in the choice and design of interventions, which is crucial for reducing the burden of malaria in Malawi.

## Background

The burden of malaria in Malawi, like other parts of sub-Saharan Africa, is a major public concern [1,2]. Recent estimates report that malaria contributes about 35% of all illnesses in children under five years of age in the country [2,3]. Current malaria control initiatives aim at halving the burden by the year 2010 through integrated control programmes encompassing vector control (via insecticide-treated nets and indoor residual spraying), intermittent preventive treatment for pregnant women and prompt and effective case management [2,4]. Effective control requires evidence-based utilisation of resources. The type and degree of interventions need to be based on epidemiological patterns of malaria risk. Malaria risk varies in space and time [5]. It is important to describe the spatio-temporal variability of malaria risk to guide control programmes [6-8].

In the last decade, maps have been produced at different geographical scales in sub-Saharan Africa [9-13], following the Mapping Malaria Risk in Africa (MARA) project [14], with the aim of identifying areas where greatest control effort should be focussed. In this analysis, the objective was to predict and map malaria risk in Malawi using point-referenced prevalence data. Existing risk maps are based on a theoretical climatic model [15] or expert opinion [2], but these have important limitations as they fail to provide insight into the transmission of malaria in Malawi. It is important to characterise malaria risk based on empirical evidence using a malaria-specific indicator, in this case, malaria prevalence of infection in children, and assess its relationship with environmental risk factors.

Prediction of risk based on point-referenced data presents some challenges when the data are sparsely distributed. Such data often exhibit autocorrelation, such that locations close to each other have similar risk. Models should allow for spatial correlation, failing which, the significance of risk factors is overstated [16,17]. Analyses of point-referenced data have been carried out using geostatistical models [18], for optimal prediction. Recently, a model-based geostatistical (MBG) approach has been applied [19]. The approach permits simultaneous modelling of related issues such as risk assessment, spatial dependence, prediction and quantification of uncertainty [20,21]. Accurate prediction of risk can further be achieved by including environmental factors likely to influence malaria transmission [9].

Several studies have shown that malaria infection is influenced by environmental factors such as temperature, rainfall, humidity and elevation. Specifically, temperature and rainfall act as limiting factors on the development of *Anopheles *mosquitoes which are the intermediate hosts in the transmission of malaria parasites [22]. In tropical settings, temperature and rainfall conditions are nearly always favourable for transmission. Humidity is also suitable for transmission because it affects the survival rate of mosquitoes. Similarly, elevation above sea level (asl) is known to define the ecology of malaria transmission through temperature [23,24]. At certain altitudes malaria transmission does not occur because of extreme temperatures that inhibit the mosquito and parasite life-cycle. For small countries like Malawi, topography remains a single most important factor that defines large-scale differences in malaria risk because climatic variables change little over the limited range of latitude.

In this study, we applied the model-based geostatistical (MBG) approach to analyse and predict malaria risk in Malawi, using point-referenced prevalence data realised from previous mass malariometric surveys carried out in the country. We adjusted for environmental covariates to accurately predict malaria risk.

## Methods

### Data sources

**Figure 1:**
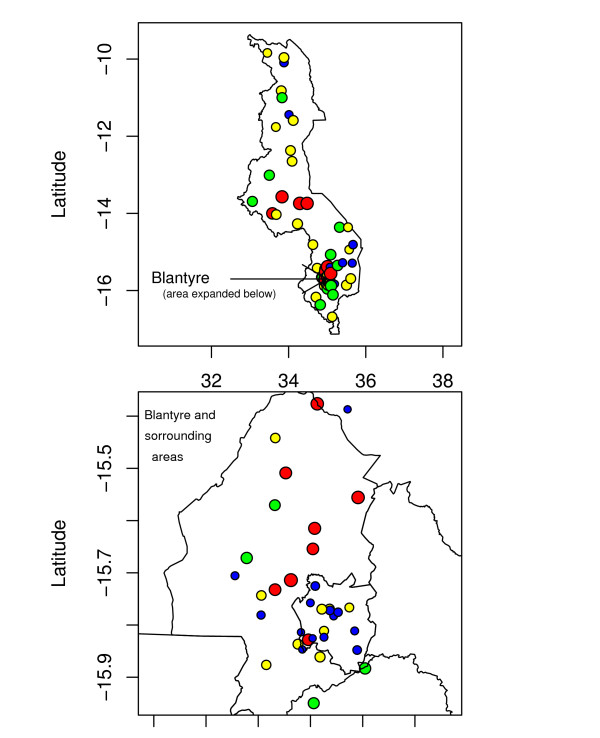
Point map showing malaria prevalence of children aged 1–10 years, at 73 locations in Malawi from 1977 to 2002. Data divided into quartile: Blue: 0–25%, Green: 26–50%, Yellow: 51–75%, Red: 76–100%.

Analysis and mapping were based on point-referenced prevalence ratio data of children aged 1–10 years, obtained at 73 survey sites across the country (Table 1 and Figure 1). For highly malaria endemic areas like Malawi, children in this age group are mostly non-immune, hence the clinical outcome of infection of this group is a good indication of transmission intensity [14,25]. Data were abstracted from grey or published literature based on collection methods outlined in MARA technical report [14]. For each data point we extracted the age-specific number of children examined and who tested positive for parasitemia, date and year when the survey was carried out, the method used to analyse the blood sample as well as the purpose for which the survey was carried out. For each site, the data of subjects aged 1–10 years were combined by taking the average across years. We included all surveys that examined at least 50 children, and locations which had smaller samples but were close (< 10 km) were combined.

**Table 1:** Observed mean, minimum and maximum prevalence ratios by districts

District^†^	Mean prevalence	Minimum prevalence	Maximum prevalence	Data points
Blantyre	0.44	0.08	0.91	31
Chikwawa	0.59	0.41	0.92	4
Chiradzulu	0.13	0.05	0.25	2
Chitipa	0.26	0.09	0.37	1
Dedza	0.50	0.20	0.68	1
Dowa	0.87	0.11	0.93	1
Karonga	0.35	0.24	0.46	2
Kasungu	0.53	0.20	0.84	1
Lilongwe	0.59	0.43	0.75	2
Machinga	0.36	0.24	0.59	3
Mangochi	0.52	0.16	0.70	1
Mchinji	0.50	0.04	0.60	1
Mulanje	0.48	0.25	0.55	2
Mwanza	0.25	0.08	0.43	3
Mzimba	0.24	0.18	0.30	2
Nkhatabay	0.46	0.22	0.74	1
Nkhotakota	0.44	0.19	0.63	2
Ntcheu	0.43	0.07	0.71	1
Rumphi	0.48	0.41	0.51	2
Salima	0.87	0.78	0.93	3
Thyolo	0.63	0.39	0.67	2
Zomba	0.25	0.05	0.57	5

^†^Districts where data were available

At each data location we also extracted the values of the following covariates: (i) mean annual maximum temperature, (ii) mean annual rainfall, (iii) mean annual potential evapotranspiration (PET), and (iv) elevation, from the Spatial Characterization Tool (SCT) [26]. The SCT is a suite of geographically referenced environmental information including those used here, for most sub-Saharan countries, and provides a framework to compile, querry and access the data. The climatic variables are interpolated gridded surfaces generated from long-term monthly means of historical weather station point data, from the period 1920–1980, across Africa. The elevation data were interpolated from digital elevation models of Africa.

### Statistical analysis

Variograms based on the empirical logit of observed prevalence were computed to explore the spatial correlation in the data. Bivariate non-spatial logistic regression models (Equation 1), were fitted for each variable of the following variables: elevation in metres asl, mean maximum temperature in degrees Celsius, rainfall in millimetres per annum and PET in millimetres per annum. Because of possible nonlinear relationships, each variable was converted into a categorical variable for further analysis, with cutoffs based on the natural break points, guided by exploratory scatterplots. The highest category of each variable was considered the reference category, and variables significant at < 0.2 were used to fit bivariate spatial logistic models (Equation 2).

Assume that the number found positive for malaria parasitemia at location *i *is *Y*_*i *_out of *N*_*i *_examined, then *Y*_*i *_is a binomial random variable, *Y*_*i *_~ *Bin *(*N*_*i*_, *p*_*i*_), where *p*_*i *_is the proportion infected at each location. The bivariate ordinary logistic model is given by

log⁡(pi1−pi)=β0+β1vi     (1)
 MathType@MTEF@5@5@+=feaafiart1ev1aaatCvAUfKttLearuWrP9MDH5MBPbIqV92AaeXatLxBI9gBaebbnrfifHhDYfgasaacH8akY=wiFfYdH8Gipec8Eeeu0xXdbba9frFj0=OqFfea0dXdd9vqai=hGuQ8kuc9pgc9s8qqaq=dirpe0xb9q8qiLsFr0=vr0=vr0dc8meaabaqaciaacaGaaeqabaqabeGadaaakeaacyGGSbaBcqGGVbWBcqGGNbWzdaqadiqaamaalaaabaGaemiCaa3aaSbaaSqaaiabdMgaPbqabaaakeaacqaIXaqmcqGHsislcqWGWbaCdaWgaaWcbaGaemyAaKgabeaaaaaakiaawIcacaGLPaaacqGH9aqpiiGacqWFYoGydaWgaaWcbaGaeGimaadabeaakiabgUcaRiab=j7aInaaBaaaleaacqaIXaqmaeqaaOGaemODay3aaSbaaSqaaiabdMgaPbqabaGccaWLjaGaaCzcamaabmGabaGaeGymaedacaGLOaGaayzkaaaaaa@4871@

where *β*_0 _is the intercept, *v*_*i *_is a covariate, *β*_1 _is the corresponding regression parameter. The spatial correlation is modelled by inclusion of a random effect *S*_*i*_, i.e.

log⁡(pi1−pi)=β0+β1vi+Si.     (2)
 MathType@MTEF@5@5@+=feaafiart1ev1aaatCvAUfKttLearuWrP9MDH5MBPbIqV92AaeXatLxBI9gBaebbnrfifHhDYfgasaacH8akY=wiFfYdH8Gipec8Eeeu0xXdbba9frFj0=OqFfea0dXdd9vqai=hGuQ8kuc9pgc9s8qqaq=dirpe0xb9q8qiLsFr0=vr0=vr0dc8meaabaqaciaacaGaaeqabaqabeGadaaakeaacyGGSbaBcqGGVbWBcqGGNbWzdaqadiqaamaalaaabaGaemiCaa3aaSbaaSqaaiabdMgaPbqabaaakeaacqaIXaqmcqGHsislcqWGWbaCdaWgaaWcbaGaemyAaKgabeaaaaaakiaawIcacaGLPaaacqGH9aqpiiGacqWFYoGydaWgaaWcbaGaeGimaadabeaakiabgUcaRiab=j7aInaaBaaaleaacqaIXaqmaeqaaOGaemODay3aaSbaaSqaaiabdMgaPbqabaGccqGHRaWkcqWGtbWudaWgaaWcbaGaemyAaKgabeaakiabc6caUiaaxMaacaWLjaWaaeWaceaacqaIYaGmaiaawIcacaGLPaaaaaa@4CF9@

The spatial component, *S*_*i*_, is assumed to be a zero mean Gaussian process with variance *σ*^2 ^and correlation function *ρ *(*d*_*ij*_, *φ*). The range *φ *measures the rate of decay of spatial autocorrelation, and *d*_*ij *_= *x*_*i *_- *x*_*j *_measures the Euclidean distance between locations *x*_*i *_and *x*_*j*_. Under the spatial model, the response *Y*_*i *_given the random effects *S*_*i *_and covariates *v*_*i*_, is assumed to be conditionally independent and distributed as a binomial outcome [19,21].

All candidate variables identified in the bivariate spatial models were included in the multiple spatial logistic model (Equation 2) for prediction. However, in the multiple model, parameter *β*_1 _becomes a vector of regression coefficients corresponding to the vector of covariates *v*_*i*_.

**Figure 2:**
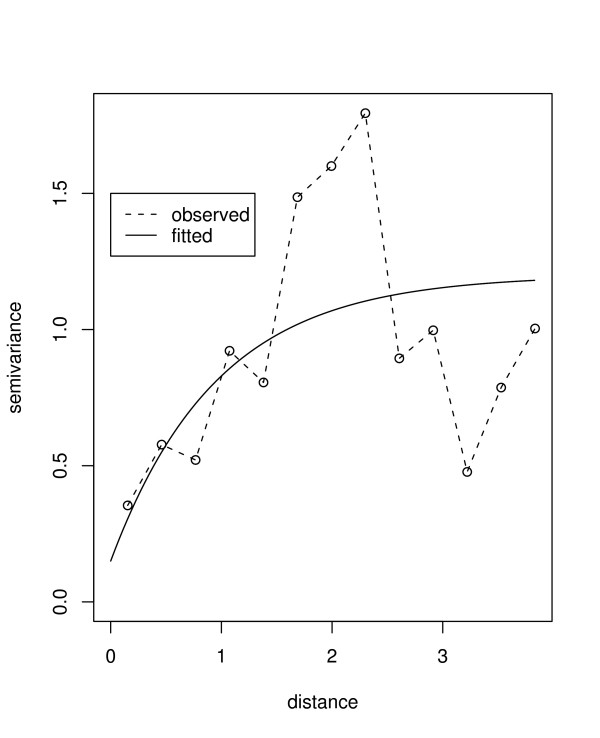
Empirical and fitted variogram of the logit transformed prevalence rate of infection. Separation distance is given in degrees latitude. Note: at equator one degree is approximately 120 km.

Model estimation was achieved using a Bayesian approach. Accordingly, all parameters in model 2 were assigned prior distributions. In Bayesian statistical inference, prior distributions are the assumptions about the distributions of the parameters [27]. In this analysis, diffuse priors were assigned to the fixed effect terms *β*_0 _and *β*_1_. For the correlation function an exponential function, exp(-*d*_*ij*_/*φ*), was assumed guided by the empirical variogram (Figure 2). The range (*φ*) and variance (*σ*^2^) were then assigned reciprocal priors, also known as Jeffreys priors, i.e., *f*(*φ*) ∝ 1/*φ *and *f*(*σ*^2^) ∝ 1/*σ*^2^. The model was implemented using geoRglm [28], a package based on the R statistical system [29]. About 90,000 Markov Chain Monte Carlo (MCMC) iterations were run, with the initial 30,000 discarded and every 20th sample stored, yielding a sample of 3,000 for assessing convergence and parameter estimation. We assessed MCMC convergence of all model parameters by examining trace plots and autocorrelation plots of the MCMC output after burn-in. A variogram of residuals was plotted to evaluate whether spatial autocorrelation was removed by the spatial model. This was assisted by computing envelopes for the variogram, using data permutations under the assumption of no correlation. If the variogram plot falls within the envelope, it means a reduction in spatial correlation has been achieved. Similar assessment was carried out by calculating Moran's I statistic based on the residuals [30].

For mapping, we predicted prevalence of infection at 4000 grid locations covering the entire country. Covariates identified as significant in the bivariate spatial model were used in prediction model. The predicted values were posterior medians realised as part of the MCMC simulations from the posterior predictive distribution [21],

*P *(*Y*_0_|*Y*) ∝ *P *(*Y*_0_|*β*, *S*_0_) *P *(*S*_0_|*S*, *σ*^2^, *φ*) *P *(*β*, *S*, *σ*^2^, *φ*|*Y*),     (3)

where *Y*_0 _are predicted values at new locations given the observed data (*Y*), and *S*_0 _is the prediction of some functional of spatial process *S*. Similarly, approximate standard errors were obtained by dividing the 95% credible interval by 4. The estimates, *Y*_0_, were then exported to ArcGIS (Version 8.3; ESRI, 2004) for cartographic representation. We overlayed the final predicted prevalence map on a population density map to calculate the population at risk for different endemicity categories.

## Results

### Spatial analysis of malaria infection

Table 1 gives descriptive summaries of observed prevalence, by district, of children between the age of 1 and 10 years. Figure 1 displays locations of points at which prevalence surveys were conducted. Relatively high prevalence of infection (> 25%) was observed for most sites. There was a high concentration of points within Blantyre district (bottom map) following the baseline survey carried out in the year 2000 by the Blantyre Malaria Initiative [3,31]. Table 2 shows the posterior medians (given as odds ratios: OR) and the corresponding 95% credible intervals (CI) for the non-spatial and spatial logistic models. In the non-spatial model, malaria risk was significantly associated with elevation, mean annual maximum temperature, PET and rainfall. Figure 2 presents variograms of logit transformed rates of infection, which clearly indicated small scale spatial autocorrelation. The high concentration of points within Blantyre was investigated to assess if this did not affect the correlation structure, by fitting variogram plots without the Blantyre data points. The behaviour depicted by the plots was similar to that of Figure 2, suggesting a stationary variogram was appropriate.

**Table 2:** Variables and regression results from the bivariate non-spatial, Bayesian bivariate and multiple spatial logistic models.

Variable	Bivariate non-spatial model		Bivariate spatial model		Multiple spatial model	
	OR^†^	(95% CI^‡^)	OR	(95% CI)	OR	(95% CI)

*Elevation*						
*β*_1_(elev1: < 650 m)	1.33	(1.21, 1.46)	1.98	(1.29, 3.05)	1.42	(1.13, 1.99)
*β*_2 _(elev2: 650–1110 m)	0.82	(0.75, 0.91)	2.08	(1.53, 2.81)	1.89	(1.38, 2.59)
Reference (elev3 : > 1110 m)	1.00		1.00		1.00	
*Max. Temperature*						
*β*_3 _(Tmax1: < 27°C)	0.77	(0.71, 0.83)	1.88	(1.52, 2.34)	1.68	(1.45, 2.73)
*β*_4 _(Tmax2: 27–32°C)	0.63	(0.59, 0.68)	0.85	(0.69, 1.02)	0.88	(0.43, 1.08)
Reference (Tmax3: > 32°C)	1.00		1.00		1.00	
*Rainfall*						
*β*_5 _(Rain1: < 880 mm)	1.15	(1.00, 1.31)	0.81	(0.64, 1.14)		
*β*_6 _(Rain2: 880–1180 mm)	1.20	(1.11, 1.29)	0.76	(0.54, 2.07)		
Reference (Rain3: > 1180 mm)	1.00		1.00			
*PET*^¶^						
*β*_7 _(PET1: < 1370 mm)	0.72	(0.61, 0.84)	0.49	(0.33, 1.22)	0.69	(0.21, 2.34)
*β*_8 _(PET2: 1370–1510 mm)	0.61	(0.58, 0.65)	0.38	(0.17, 0.89)	0.41	(0.14, 0.97)
Reference (PET3: > 1510 mm)	1.00		1.00		1.00	

Range (*φ*)					0.54	(0.23, 0.96)
Variance (*σ*^2^)					13.74	(8.80,20.16)

^†^OR = Odds ratio

^‡^CI = Credible interval

^¶^PET = Potential evapotranspiration

Results from the bivariate spatial logistic models are given in Table 2. Overall, elevation, mean maximum temperature and PET were associated with malaria prevalence after adjusting for spatial correlation. At elevation of < 650 m asl, relative to elevation ≥ 1110 m, the risk of malaria was higher (OR: 1.98, 95% CI: 1.29–3.05). At elevation between 650 m and 1100 m asl, relative to elevation ≥ 1110 m, there was increased malaria risk (OR: 2.08, 95% CI: 1.53–2.81). The risk of malaria was likely to be more at mean annual maximum temperature of < 27°C relative to temperature > 32°C (OR: 1.88, 95% CI: 1.52–2.34). At temperatures between 27–32°C, relative to temperature > 32°C, the risk was less (OR: 0.85, 95% CI: 0.69–1.02). Rainfall of less than 800 mm and between 800–1180 mm per annum relative to rainfall of more than 1180 mm was not associated with malaria prevalence (OR: 0.81, 95% CI: 0.64–1.14 and OR: 0.76, 95% CI: 0.54–2.07 respectively). Similarly, PET of less than 1370 mm compared to PET of > 1510 mm was not associated with malaria risk (OR: 0.49, 95% CI: 0.33–1.22). However, PET between 1370–1510 mm was significantly associated with lower prevalence of infection than PET levels over 1510 mm (OR: 0.38, 95% CI: 0.17–0.89).

**Figure 3:**
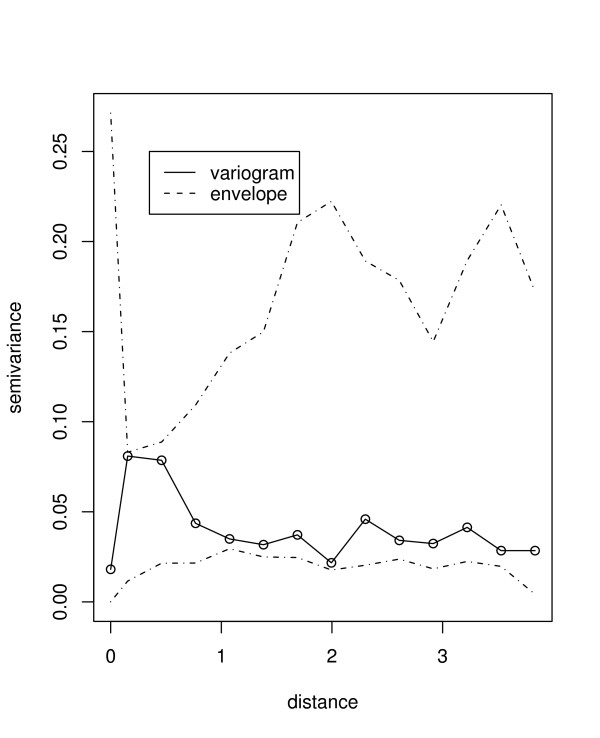
Empirical variogram from standardized Pearson residuals (solid line) realized from the final spatial model, with a simulated envelope for the variogram (dotted lines). Separation distance is given in degrees latitude. Note: at equator one degree is approximately 120 km.

The multiple model included elevation, mean maximum temperature and PET. This model was used for prediction of malaria risk in places where data were not observed. Results of the final model and the spatial correlation parameters obtained are given in the Table 2. Again elevation was associated with the spatial distribution of malaria risk. However, maximum temperature and PET were marginally associated with malaria risk. The practical range of correlation at which observations were uncorrelated with increasing distance was 3 × 0.54 = 1.62 degrees latitude (approximately 179.8 km, with 95% interval: 76.6–299.7 km). The variance of spatial heterogeneity was 13.74 (95% interval: 8.8–20.2). Figure 3 shows the plot of the variogram of the residuals, which indicates some considerable reduction in spatial correlation since all points fall inside the variogram envelope. This is also apparent from Moran I statistics (*I *= -0.92, *p *– *value *= 0.36) which indicates no significant residual spatial correlation.

### Maps and population at risk

**Figure 4:**
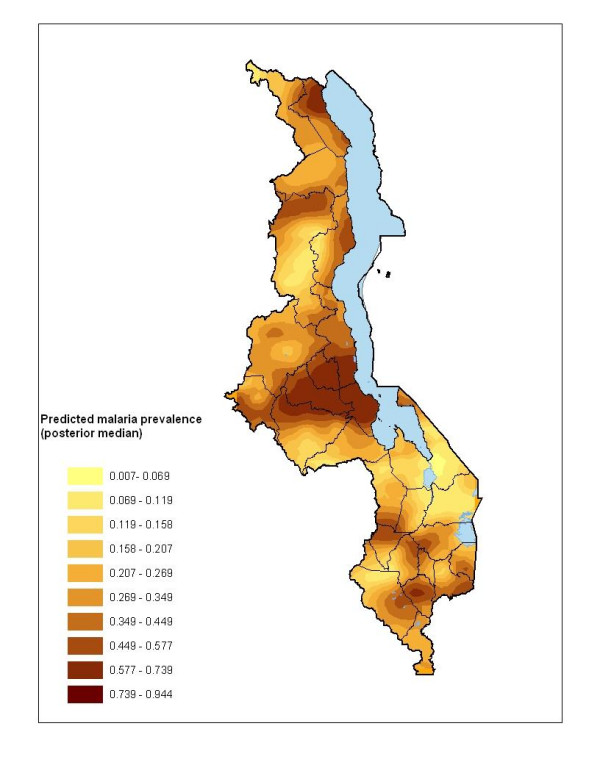
Map showing predicted prevalence rate based on the posterior median of the predictive distribution with district boundaries and major water bodies (in blue). Cartographic visualization was carried out in ArcGIS.

**Figure 5:**
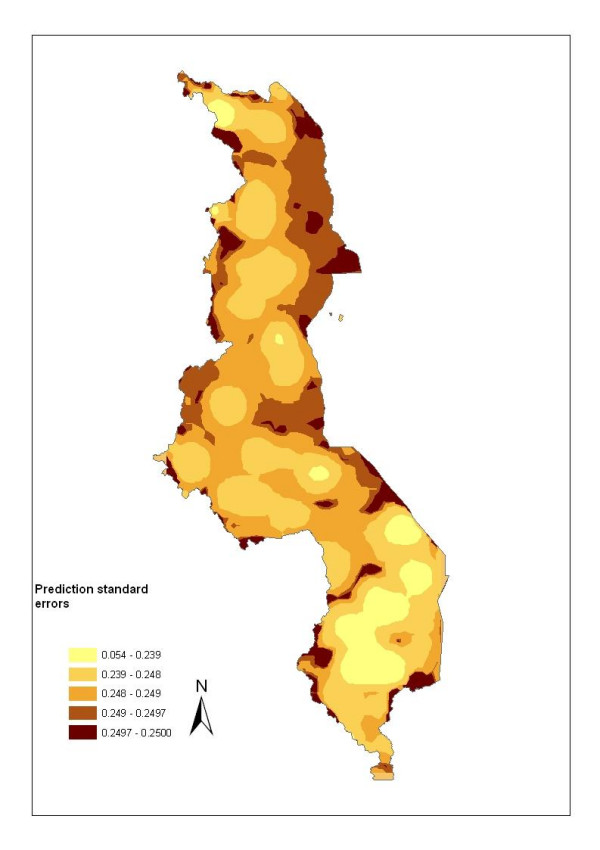
Map showing the prediction standard errors which are useful to quantify map precision. Cartographic visualization was carried out in ArcGIS.

The map of predicted prevalence is given in Figure 4. The predicted prevalence ranged from 0.7% to 94%. Relatively higher risk areas were predicted in the central and northern region districts as well as along the lakeshore districts on the east central side of the country. Other notable areas with relatively higher risk were in the south-western region (parts of Ntcheu, Zomba, Mwanza and Balaka districts). Low rates of between 0.7–16% were predicted around the southern region over the highland ranges of Zomba, Blantyre, and Mwanza and parts of Chikwawa. Other areas with low rates were on the north-western regions, for instance, the districts of Mzimba, Rumphi and Chitipa. These areas are predominantly at high altitude (l,260–2,400 m asl). Figure 5 shows errors of estimation which ranged from 0.05 to 0.25. As one would expect, high error values were observed away from the data locations, while small errors were obtained closer to the data locations. Blantyre and surrounding areas had small errors due to relatively large number of data points available for model estimation.

Table 3 gives the estimated population at risk of children ≤ 10 years in each district by risk category. The overall at-risk population was estimated at 2,732,434 based on 2005 population projections [32]. The risk distribution was 14, 22, 25, 12 and 27% for the risk categories 0–15%, 16–30%, 31-5%, 46–60% and 61–100% respectively. About 1 million (39%) children lived in the medium to high risk areas (46–100%), while approximately 27% lived in highly endemic areas (61–100%). Moreover, there were marked differences in population at risk between districts. Most at-risk population were living either on the central plain or along the lake shore of the country.

**Table 3:** Predicted percentage of at-risk population of children aged 1–10 years, by district and risk category.

District	0–15%	15–30%	30–45%	45–60%	60–100%	Population^† §^
Likoma	0	0	100	0	0	1,173
Chitipa	30	40	20	10	0	21,098
Karonga	0	20	10	10	60	31,267
Rumphi	10	60	20	5	5	20,531
Mzimba	50	20	15	15	0	97,263
Nkhatabay	20	10	20	20	30	24,380
Mzuzu City	0	70	30	0	0	12,627
Nkhotakota	0	15	15	20	50	38,184
Ntchisi	0	0	0	5	95	27,166
Dowa	0	0	0	5	95	67,357
Salima	0	0	0	5	95	41,126
Kasungu	0	10	30	30	30	78,357
Mchinji	0	0	20	20	60	53,843
Lilongwe rural	20	20	15	15	30	214,472
Lilongwe City	0	25	25	25	25	63,557
Dedza	15	20	60	5	0	81,928
Ntcheu	40	40	15	5	0	58,214
Mangochi	40	25	25	5	5	99,604
Balaka	30	30	30	5	5	39,273
Machinga	25	20	25	10	20	60,757
Chiradzulu	30	30	20	20	0	34,236
Phalombe	0	0	15	15	75	35,682
Mulanje	5	15	30	20	30	61,682
Blantyre rural	20	40	30	10	0	112,095
Blantyre city	0	85	15	0	0	67,009
Zomba	0	10	70	10	10	81,298
Zomba city	0	10	10	15	65	9,143
Mwanza	40	25	15	20	0	22,341
Chikwawa	15	15	20	20	30	57,355
Thyolo	0	5	35	10	50	68,442
Nsanje	10	30	60	0	0	32,248

Total	14	22	25	12	27	2,732,434

^†^Population estimates at mid-year 2005

^§^Source: National Statistics Office [32]

## Discussion

In this analysis, a map showing the spatial variation of malaria risk in children aged 1–10 years in Malawi was produced using point-referenced prevalence of infection data. The map is a first attempt towards the empirical description of malaria risk in Malawi, and differs from the climatic suitability model map [15] or expert opinion [2]. In contrast to expert's broad classification of malaria risk, this map (Figure 4) shows that malaria risk varies widely in the country even within districts. Nevertheless, in agreement with expert opinion [2,31,33], our map identifies highest risk along the lakeshore, Shire river valley and central plain areas, and lowest in the highland areas of Rumphi, Mzimba, Chitipa and the Kirk range.

The map will be useful for focussed malaria control activities. Currently, for example, there are plans to scale-up the coverage of insecticide treated nets (ITN) as part of the strategy to reduce the burden of malaria [34]. It is important, therefore, to identify and carefully plan prior to scaling-up the ITN program. Among other things, this map can advise areas to be targeted, for instance all areas at highest risk. Considering the at-risk population estimates (Table 3), and the recent cost estimates of net delivery [35], the cost of scaling-up can be calculated. Since estimates are available up to the local level, it is possible to plan up to that scale. Furthermore, the estimates may provide baseline information against which the success of an intervention programme could be assessed, through follow-up surveys in the future. A case in point is the Innovative Vector Control Consortium which plans to extend insecticide residual spraying, currently implemented in Mozambique, to southern Malawi [36]. The effectiveness of their tools can be compared against this map. Sentinel points for spraying can be selected using this product. Another control initiative that may find the map useful is the President's Malaria Initiative (PMI), which is a U.S. government initiative designed to cut malaria deaths in half in target countries in sub-Saharan Africa, including Malawi [37].

The analysis shows the importance of integrating risk factors in the spatial prediction of malaria risk. Generally, the results showed that elevation plays an important role in defining malaria risk in Malawi, a fact recognized by experts in the country [31,33], and has been confirmed in several other studies carried out in the continent [23,24,38]. The fact that malaria prevalence was only marginally associated with climatic variables can be explained by the limited range of latitudes within small countries like Malawi, and hence the minimal variation in climate. The results, therefore, do not contradict the importance of climatic variables in predicting malaria risk in general [15,22].

The modelling was based on spatial statistical methods. These offer an attractive and better alternative to the GIS mapping approach which incorporates the spatial correlation inherent in the data [9,17,19,20]. Furthermore, the method allows errors of estimations to be quantified making it possible to assess the precision of the map and significance of covariates (Figure 5). In addition, adjusting for spatial correlation avoids overstating the significance of covariates (Table 2) [16,17]. Spatial correlation may arise because of omitted or unobserved covariates, and incorporating the spatial random effect in the model further allows these to be accounted for [39].

The results presented here have some limitations. First, the data points used for analysis were available at 73 sites, but were sparsely distributed in the northern and central region (Figure 1). This has potential to bias the estimates. However, the inclusion of risk factors at predicted sites may have reduced this bias. Second, the data used here span a period of 20 years, and the risk may have not been constant with time. For example, increase in population density, urbanization, agricultural and socio-economic development have brought change over this period, which may have affected the pattern of malaria risk. Be that as it may, the high endemicity and the absence of sustainable and effective interventions in the country suggest that malaria risk has changed little during this time. Accordingly, such data can still be used to generate reliable and informative malaria risk estimates. Another limitation is that the age range of 1–10 years may not be ideal as the level of immunity may not be homogenous in this age group. Gemperli *et al *[11] provide a method of converting a set of heterogeneous age-specific prevalence onto a common scale of transmission intensity for prediction and mapping purposes.

Malaria transmission is very complex and prediction based on few covariates may compromise the accuracy of the map. Malaria transmission drivers go beyond topographical and climatic variables, and may include socio-demographic factors and include urbanization, population growth and local variation in vector habitat [40-42]. In practice, a wide range of environmental covariates have been used [12]. When as many independent covariates as possible are added to the model, the accuracy of the predicted map may be maximized and it may be worthwhile to explore how the map would change when relevant covariates become available. Updating malaria maps should therefore be carried out on a regular basis as new data become accessible.

Despite these limitations, the map of predicted risk of infection provides a much needed characterization of geographical variation of malaria risk in Malawi. It is the only one that provides estimates at all locations, and therefore offers much needed evidence-based stratification of malaria risk. Through the map it is possible to determine which areas require greatest control effort. More important, it provides a baseline against which the effectiveness of current control efforts can be assessed.

## Abbreviations

CI Confidence Interval; Credible Interval

GIS Geographical Information Systems

ITN Insecticide treated nets

MARA Mapping Malaria Risk in Africa

MBG Model based geostatistics

MCMC Markov Chain Monte Carlo

OR Odds Ratio

SCT Spatial Characterisation Tool

## Authors' contributions

LNK conceptualized, collected data, analyzed and drafted the manuscript. IK and BLS participated in the conception, and critical review of the manuscript. THH participated in data collection and critical review of manuscript.
